# Biochemical recurrence-free survival and pathological outcomes after radical prostatectomy for high-risk prostate cancer

**DOI:** 10.1186/s12894-016-0146-6

**Published:** 2016-06-08

**Authors:** Jean-Baptiste Beauval, Mathieu Roumiguié, Thomas Filleron, Thibaut Benoit, Alexandre de la Taille, Bernard Malavaud, Laurent Salomon, Michel Soulié, Guillaume Ploussard

**Affiliations:** Department of Urology, Andrology and Renal Transplantation, CHU Rangueil, 1, av J Pouilhès, 31059 Toulouse, France; Institut Claudius Regaud, IUCT-O, Toulouse, F-31059 France; Department of Urology, Andrology and Renal Transplantation, CHU Mondor, Créteil, France; Department of Urology, Clinique St Jean du Languedoc, Toulouse, France

**Keywords:** High risk prostate cancer, Radical prostatectomy, BCR free-survival, Risk factors, Stratification

## Abstract

**Background:**

We propose to improve the prognostic assessment after radical prostatectomy (RP) by dividing high-risk prostate cancer (hrPCa) (according to the d’Amico classification) into subgroups combining 1, 2 or 3 criteria of aggressiveness (cT2c-T3a, PSA >20 ng/ml, Gleason score (GS) > 7).

**Methods:**

Data from 4795 hrPCa patients who underwent RP in two French university hospitals from 1991 to 2013 were analyzed. Subgroups were formed to determine whether an increasing number (1, 2 or 3) of criteria of tumor aggressiveness was associated with poorer oncological results and early biochemical recurrence (BCR) (PSA > 0.2 ng/ml). These results were compared using Fisher’s exact test and BCR was compared according to the Kaplan-Meier method.

**Results:**

Eight hundred fifteen patients were treated by RP for hrPCa (8 %). Four hundred eleven patients (79.5 %) presented 1 RF (Risk Factor), 93 (18.0 %) 2 RF and 13 (2.5 %) 3 RF. Lymph node invasion and positive margin rates were 12.4 and 44.1 %, respectively. The prognostic sub-stratification based on these 3 factors was significantly predictive for adverse pathologic features and for oncologic outcomes. BCR free survival was respectively 56.4, 27.06 and 18.46 % for 1RF, 2RF and 3RF (*p* < 0.0001). However, no predominant negative criterion was found.

**Conclusion:**

Oncologic results after RP are heterogenous within the hrPCa risk group. Sub-stratification based on three well-defined criteria leads to a better identification of the most aggressive cancers. On the other hand, RP provides both effective cancer control and satisfactory survival rates in patients with only one risk factor.

**Electronic supplementary material:**

The online version of this article (doi:10.1186/s12894-016-0146-6) contains supplementary material, which is available to authorized users.

## Background

Prostate cancer (PCa) is the most common form of malignant cancer in Europe and, the second leading of death attributable of cancer [1, 2]. Despite the widespread use of prostate specific antigen (PSA) individual screening, some patients are still diagnosed with locally advanced and/or high risk PCa. According to the D’Amico’s classification, patient with PSA > 20 ng/mL and/or preoperative Gleason score of 8–10 and/or clinical stage ≥ T2c can be considered to be at high-risk of disease progression despite radical treatment with a curative intent [3].

In high-risk prostate cancer patients, the best course of treatment is often unclear, and the oncological outcomes appear heterogeneous among series and treatment options. Even though several treatment options, including RP, RT, and androgen deprivation therapy (ADT) alone or in combination, are available but the recurrence rate remains high regardless of the type of treatment [4]. Recently, long-term follow-up studies of high-risk PCa patients who underwent RP with or without adjuvant therapies have revealed good oncologic outcomes highlighting the potential underutilization of surgery in such cases [5, 6]. Retrospective population-based studies recently suggested that oncologic outcomes in terms of disease-specific mortality were at least comparable in high risk PCa patients treated with RP as compared to those undergoing radiotherapy combined with androgen deprivation therapy [6–8]. Evidence also suggests that patients with high-risk PCa are those who benefit the most from RP [8–10].

Thus, large multicentric series have reported that a significant percentage (about 30 %) of PCa preoperatively defined as high risk, was organ-confined and favourable in RP specimens [11–13]. These findings highlight the interest of improving patients selection within the heteregeneous group of high risk PCa.

The aim of this study was to define subgroups combining 1, 2 or 3 criteria of aggressiveness (cT2c-T3a, PSA > 20 ng/ml, Gleason score (GS) > 7) among surgically treated hrPCa and to define their risk of progression.

## Methods

### Patient sample

After institutional review board approval (patient records/information were anonymized and de-identified prior to analysis, consent was not required for your study), we retrospectively examined data from 815 consecutive patients who underwent radical prostatectomy and bilateral extended pelvic nodes dissection (in 98.1 % cases) for clinical high-risk prostate cancer in D’Amico risk classification (PSA >20 ng/ml, clinical T2c or more stage, biopsy Gleason sum 8–10) between 1990 and 2013 in two French academic centers (overall cohort: 4795 RPs). Surgical procedures were performed by 7 different senior surgeons, who used standardized techniques (open, laparoscopic or robot-assisted RP) and applied the same anatomic template during pelvic lymph node dissection, as previously described [14]. We excluded 298 men because of incomplete information on preoperative PSA, Gleason score, clinical stage and pathologic T stage. Only patients with complete clinical and pathological data who did not receive neoadjuvant therapies were eligible. In the final analysis, the data on the 517 patients included the preoperative parameters such as age, prostatic specific antigen (PSA), clinical stage (CS) and biopsy Gleason score.

The clinical stage was assigned according to the 2002 TNM staging system, prostate biopsy cores were obtained with transrectal ultrasound guidance, using a >10-core biopsy protocol, and pretreatment PSA was measured before digital rectal examination. Genitourinary pathologists assessed the biopsy and pathologic gradings according to the Gleason gradings system before 2005 and the modified ISUP Gleason score after 2005. The pT stage was graded according to the 2002 AJCC staging system for PCa.

Biochemical recurrence (BCR) was defined as a PSA value 0.2 ng/ml after RP, confirmed by at least two consecutive measurements.

### Statistical analysis

Data were summarized by frequency and percentage for categorical variables, and by median and range for continuous variables. Comparisons between groups were performed using the Mann–Whitney rank sum test for continuous variables and Chi square or Fisher’s exact test for categorical variables.

Biochemical Recurrence Free Survival was calculated from the date of the surgery to the date of the diagnosis of the biochemical recurrence. BCR was estimated using Kaplan-Meier method and univariate analysis was performed using the log rank test. Logistic regression models were built to determine the independent predictive value of PSA, Gleason score and clinical stage at diagnosis.

All statistical tests were two sided, and differences were considered statistically significant when *p* < 0.05. Stata 13.0 software (StatCorp LP, College Statio, Texas) was used for all statistical analysis.

## Results

Patients’ descriptive characteristics are to be found on Table 1. 411 patients (79.5 %) presented 1 RF (Risk Factor), 93 (18.0 %) 2 RF and 13 (2.5 %) 3 RF. Median serum PSA level was 21.0 (1.7:158.0) ng/ml. According to the risk classification, median PSA was 15.7 (1.7-158.0) for 1RF, 30.0 (3.0-134.0) for 2RF, 31.7 (22.0-50.0) for 3RF, respectively (*p* < 0.001). The proportions of the clinical stage were different between the 3 groups: cT1c-cT2b: 91.2 % (*n* = 375), 35.5 % (*n* = 33) and 0 %; cT2c-cT3: 8.8 % (*n* = 36), 64.5 % (*n* = 60) and 100 % (*n* = 13)) respectively for 1RF, 2RF and 3RF (*p* < 0.0001). Furthermore, the proportions of Gleason sum >7 found into prostate biopsy was greater in population with less RF (43.6 % (*n* = 179), 62.4 % (*n* = 58) and 100 % (*n* = 13) respectively for 1RF, 2RF and 3RF (*p* < 0.0001). In overall population, the pT stage was pT2 in 29 % of patients, pT3a in 37.9 %, pT3b in 32.9 %, and pT4 in 0.2 %. Positive surgical margin was reported in 44.1 % of cases (*n* = 228) and increased within the risk sub-stratification: 40.6, 58.1, and 53.8 % for 1RF, 2RF and 3RF respectively (*p* < 0.0007)). Lymph node metastasis was noted in 12.4 % of patients: 9.2 % (*n* = 37), 22.6 % (*n* = 21) and 38.5 % (*n* = 5) for 1RF, 2RF and 3RF respectively (*p* < 0.0001). The number of nodes removed was exactly the same for the 3 groups of patients (median 10 LN (1–39). The majority of the patients had only one criterion of aggressiveness (79.5 %) (Additional file 1: Table S1).

**Table 1:** Descriptive statistics of 523 patients with clinical high-risk prostate cancer treated with radical prostatectomy and pelvic node dissection

	Overall	1RF	2RF	3RF	*p*
No (%)	523	411 (79.5)	93 (18.0)	13 (2.5)	
Missing	3				
Age,yr					
Age,yr Median	64.0	64.0	64.0	59	0.02
Range	41.0:79.0	44.0:79.0	44.0:76.0	47.0:70.0	
PSA, ng/ml					
Median	21.0	15.7	30.0	31.7	<0.001
Range	1.7: 158.0	1.7:158.0	3.0:134.0	22.0:50.0	
Clinical stage					
cT1	266 (51.5)	246 (59.9)	20 (21.5)	0 (0)	<0.0001
cT2	193 (37.3)	150 (36.5)	39 (41.9)	4 (30.8)	
cT3	58 (11.2)	15 (3.6)	34 (36.6)	9 (69.2)	
Biopsy Gleason sum					
<=7	267 (51.6)	232 (56.4)	35 (37.6)	0 (0)	<0.0001
>7	250 (48.4)	179 (43.6)	58 (62.4)	13 (100)	
Specimen Gleason sum					
<=7	301 (58.2)	250 (60.8)	50 (53.8)	1 (7.7)	0.0004
>7	216 (41.8)	161 (39.2)	43 (46.2)	12 (92.3)	
Surgical margin					
R0	289 (55.9)	244 (59.4)	39 (41.9)	6 (46.2)	0.0072
R1	228 (44.1)	167 (40.6)	54 (58.1)	7 (53.8)	
Pathological stage					
pT2	150 (29.0)	134 (32.6)	14 (15.1)	2 (15.4)	0.0007
pT3	366 (70.8)	277 (67.4)	78 (83.9)	11 (84.6)	
pT4	1 (0.2)	0 (0)	1 (1.1)	0 (0)	
Lymph node involvement					
No	444 (87.6)	364 (90.8)	72 (77.4)	8 (61.5)	0.0001
Yes	63 (12.4)	37 (9.2)	21 (22.6)	5 (38.5)	

Data were stratified in three groups of risk factors

Second-line treatments were adjuvant treatments before recurrence in only 29 cases as follows (5.6 %): radiotherapy in 1.5 % of cases, androgen deprivation therapy in 2.1 % of cases, and RT combined with ADT in 1.7 % of cases. Salvage treatments (182 cases, 35.2 %) were salvage radiotherapy in 18 % of cases, androgen deprivation therapy in 11.8 %, RT combined with ADT in 0.8 % and chemotherapy in 4.5 %.

Overall, median follow-up was 25.2 months. The 2-year and 5-year RFS rate was 55.21 (49.80; 60.28) and 41.67 (35.35; 47.86) respectively.

**Fig. 1:**
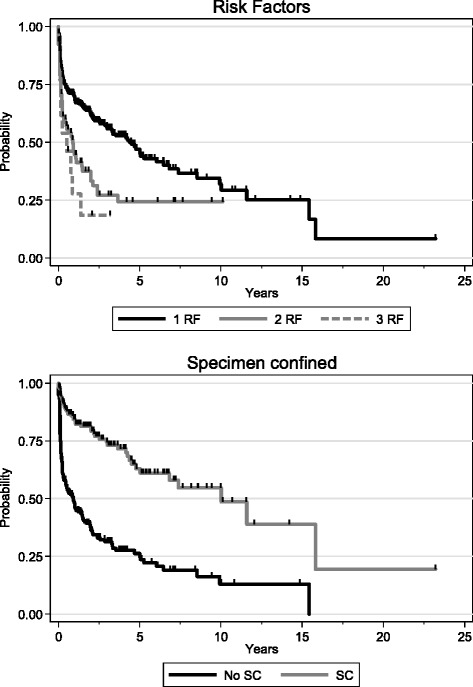
Kaplan-Meier curves depending Biochemical free recurrence rates for 3 groups of risk factors (RF)

The prognostic sub-stratification based on these 3 factors was significantly predictive for adverse pathologic features and for oncologic outcomes. BCR-free survival was, respectively, 56.4 % (50.0; 62.5), 27.06 % (16.4; 38.86) and 18.46 % (3.06; 30.33) for 1RF, 2RF and 3RF (*p* < 0.0001) (Fig. 1). However, no predominant negative criterion was however found.

In univariable analysis, predictors of oncologic outcomes were PSA, number of risk factor, postoperatively positive lymph node, surgical margin, pT stage (Table 2).

**Table 2:** Univariate analysis of biochemical recurrence (BCR)

	Event (BCR)	Survival (%)	CI 95 %	*p*
Age				
<65y	115/285	48.54	40.88; 55.77	0.8
≥65y	94/232	50.84	42.09; 58.93	
Clinical stage				
≤cT2a-b	165/408	50.44	44.04; 56.50	0.91
≥cT2c-T3	44/109	45.20	31.83; 57.64	
Biopsy Gleason score				
≤7	107/267	50.58	42.59; 58.02	0.73
>7	102/250	48.66	40.35; 56.45	
PSA				
<20	75/240	61.21	52.20; 69.02	0.0001
≥20	134/277	40.00	32.79; 47.10	
Specimen confined				
No SC	159/291	31.30	24.61; 38.19	0.0001
SC	50/226	74.41	65.75; 81.19	
Margin				
R0	82/289	66.18	58.42; 72.83	0.0001
R1	127/228	28.64	21.32; 36.36	
Risk factors				
1	148/411	56.54	50.02; 62.55	0.0001
2	51/93	27.06	16.40; 38.86	
3	10/13	18.46	3.06; 44.09	
Positive lymph node				
N0	170/453	54.14	48.00; 59.87	0.0001
N1	39/63	9.95	1.15; 30.33	
Pathological stage				
pT2	28/150	79.63	69.84; 86.54	0.0001
pT3-pT4	181/367	37.78	31.27; 44.27	

In multivariable analysis, no preoperative predictor was independently predictive for recurrence-free survival. Postoperative pT stage and positive lymph node status were independent predictors of recurrence-free survival (Table 3).

**Table 3:** Multivariate analysis of biochemical recurrence BCR

	HR	95 % CI	*p*-value
PSA preoperative			
<20 ng/ml	1	(0.99; 1.81)	0.060
≥20 ng/ml	1.34		
SC			
No	1	(0.37; 1.00)	0.051
Yes	0.61		
Surgical margin			
M-	1	(0.97; 2.17)	0.068
M+	1.45		
Number of risk factors			
1	1	(0.86; 1.70)	0.27
2	1.21	(0.70; 2.74)	0.34
3	1.38		
Lymph node invasion			
No	1	(1.46; 3.20)	<0.001
Yes	2.16		
PT Stage			
pT2	1	(1.43; 3.48)	<0.001
pT3-4	2.23		

Figure 1 (Additional file 2: Figures S1 and S2) shows Kaplan-Meier curves depending Biochemical free recurrence rates according to number of risk factors (RF), SC disease, surgical margin, lymph node invasion and stage after RP (Additional files 1 and 2). The rate of BCR free recurrence was significantly improved with the number of risk factors (log-rank test, *p* < 0.001).

## Discussion

In this current study, we demonstrated the different outcomes in high risk PCa according to the number of preoperative risk factors identified: the increasing number of RF was correlated with poorer BCR-free survival.

Indeed, at present, urologists face the dilemma of deciding which treatment is best adapted for hrPCa patients: ADT, radiotherapy, RP or a multimodality approach. When asked, most physicians commonly say they prefer ADT associated with radiotherapy [15]. Nevertheless, RP has, however, whether combined with or without multimodal therapies, produced good oncologic outcomes in the large multicentric series [11–13]. Post-surgery recurrence risk depends mainly on the pathological final assessment in RP specimens. But, an accurate preoperative patient selection is essential when choosing what initial treatment will be used in decision-making.

Preoperatively, the identification of high-risk PCa can be based on at least three well-defined predictors of the extent of the disease and the post treatment outcome as defined by d’Amico et al. [3]. Others studies and our own have shown that the D’Amico classification is a highly heterogeneous PCa risk subgroup. Because of a lack of uniform definition of hrPCa, there is an urgent need to classify hrPCa according to different prognosis groups. Indeed, the present study clearly identified 3 different populations with different oncologic outcomes, all of which depend on the number of risk factors.

The first population identified, characterized by 1 risk factor and benefited the most from surgery because of favorable cancer control with good BCR-free survival rates. Similarly, prior studies have attempted to better stratify patients with hrPCa based on the number of risks factors [12, 16]. Joniau et al. and Spahn et al. showed more favorable outcomes (BCR-free survival, CSS and OS) when there is only one. Our study confirms these results.

Conversely, the risk of recurrence appears to be very high in patients with high-grade disease and who still have at least 2 risk factors despite radical surgery along with a great deal of adjuvant and salvage treatment strategies. Multimodal treatment strategies are probably warranted in those cases. Prospective trials on the timing of adjuvant or salvage ADT and RT will hopefully provide some answers.

In our study, the PSM rate was around forty percent and more then 70 % had pT3 disease, but only 1.5 % of the patients received adjuvant radiotherapy (ART). On the other hand, salvage treatments (35.2 %) were often proposed. Most of recommendation proposed RT after RP in cases of extracapsular extension, GS > 7, PSM because of a high risk of local recurrence. Currently, the question about the time of treatment is always open: immediate adjuvant RT or salvage RT after biological monitoring. 3 studies showed a benefit of immediate ART (SWOG 8794, ARO and EORTC 22911) in comparison with no adjuvant treatment. But the time of treatment is still discussed and we should wait the results of AFU-GETUG 17 trial. Some recent studies showed a better functional recovery if RT is not performed immediately after RP. For these two reasons, urologists in this study preferred salvage RT.

Finally, we have demonstrated that men with only one high risk factor had a better BCR-free survival rate than men with two or more.

Therefore, we identified a subgroup, for which RP led to favorable outcomes, corresponding to the ideal candidate for RP in high risk PCa. Recently, Fossati et al. showed an increased diagnosis of localized and less extensive high-grade prostate cancer was observed over the last two decades. In this context, patients with high-risk disease selected for radical prostatectomy had better cancer control over time [17].

The nomogram published by Briganti et al. has led to selection improvement for patients candidates for RP as a primary treatment for high risk PCa [18]. This predictive tool helps in predicting a specimen-confined (SC) disease in clinical high risk PCa (40 % in their study). This nomogram was recently externally validated by Roumiguié et al. who confirmed good oncologic outcomes in this heterogeneous subgroup of high risk PCa thanks to a large proportion of specimen-confined PCa, but with a decreased accuracy for intermediate risks requiring improvements in treatment decision-making by new parameters such as multiparametric MRI and biomarkers [19].

Our study is not without certain limitations. Firstly, though the study period frame was long, it featured (ranging from 1990 to 2013) and with only 523 patients, sub group analysis based on years of surgery was not statistically achievable. Secondly, the changes over time in pathology assessment (Gleason score grading), patient selection and operative techniques such as nerve-sparing procedures impacting the margin status, presented unavoidable biases. No centralized pathology was available between the two centres. Finally, because of the lack of key data, the final analysis only included 523 of the 814 patients identified.

In multivariate analysis, Gleason score and number of RF was not an independent factor as showed in other studies [12, 18]. We can explain this result by a number of “poor” high-risk patients in your study to small to show statistically differences.

Finally, it is surgery (RP) itself that provides the most accurate knowledge of the pathologic tumour and to adapt adjuvant therapies based on the risk of recurrence (pT stage and lymph node invasion).

Thus, the use of such a sub-stratification prognostic approach should improve the accuracy of predicting pathologic and oncologic results, thus optimizing the selection of patients for whom primary cancer control is possible. However, even when optimal predictions are made, those high-risk patients showing adverse pathologic outcomes should still be considered as candidates for a multimodal, combined approach. In these cases, cancer control after surgery should be optimized by either adjuvant RT, HT, or a combination of both treatments.

## Conclusion

Finally, oncologic results after RP are heterogenous within the hrPCa risk group. Sub-stratification based on three well-defined criteria leads a better identification of the most aggressive cancers. In contrast, the presence of a single RF seems to be the most appropriate circumstance for RP. Such results may help urologists in scheduling post-operative monitoring and management.

## Abbreviations

ADT, androgen deprivation therapy; BCR, biochemical recurrence; GS, Gleason score; hrPCa, high risk prostate cancer; HT, hormo; LNI, Lymph node involvement; PCa, Prostate cancer; PSA, prostate specific antigen; RF, risk factor; RP, radical prostatectomy; RT, radiation therapy; SC, specimen confined

## Additional files

Additional file 1: Table S1. Different groups of risk factors (RF). (DOCX 45 kb) Additional file 2: Figures S1. Kaplan-Meier curves depending Biochemical free recurrence rates (independant factors (1)). Figure S2 Kaplan-Meier curves depending Biochemical free recurrence rates ((independant factors (2)). (DOCX 62 kb)

## References

[1] Heidenreich A, Bellmunt J, Bolla M (2011). EAU guidelines on prostate cancer. Part 1: screening, diagnosis, and treatment of clinically localised disease. Eur Urol.

[2] Bosetti C, Bertuccio P, Chatenoud L, Negri E, La Vecchia C, Levi F (2011). Trends in mortality from urologic cancers in Europe, 1970–2008. Eur Urol.

[3] D’Amico AV, Whittington R, Malkowicz SB (1998). Biochemical outcome after radical prostatectomy, external beam radiation therapy, or interstitial radiation therapy for clinically localized prostate cancer. JAMA.

[4] Garzotto M, Hung AY (2010). Contemporary management of high-risk localized prostate cancer. Curr Urol Rep.

[5] Gerber GS, Thisted RA, Chodak GW (1997). Results of radical prostatectomy in men with locally advanced prostate cancer: multi-institutional pooled analysis. Eur Urol.

[6] Sooriakumaran P, Nyberg T, Akre O (2014). Comparative effectiveness of radical prostatectomy and radiotherapy in prostate cancer: observational study of mortality outcomes. BMJ.

[7] Westover K, Chen MH, Moul J (2012). Radical prostatectomy vs radiation therapy and androgen-suppression therapy in high-risk prostate cancer. BJU Int.

[8] Abdollah F, Schmitges J, Sun M (2012). Comparison of mortality outcomes after radical prostatectomy versus radiotherapy in patients with localized prostate cancer: a population-based analysis. Int J Urol.

[9] Wilt TJ, Brawer MK, Jones KM (2012). Radical prostatectomy versus observation for localized prostate cancer. N Engl J Med.

[10] Bill-Axelson A, Holmberg L, Ruutu M (2011). Radical prostatectomy versus watchful waiting in early prostate cancer. N Engl J Med.

[11] Ploussard G, Masson-Lecomte A, Beauval JB (2011). Radical prostatectomy for high-risk prostate cancer defined by preoperative criteria: oncologic follow-up in national multicenter study in 813 patients and assessment of easy-to-use prognostic substratification. Urology.

[12] Spahn M, Joniau S, Gontero P (2010). Outcome predictors of radical prostatectomy in patients with prostate-specific antigen greater than 20 ng/ml: a European multi-institutional study of 712 patients. Eur Urol.

[13] Loeb S, Schaeffer EM, Trock BJ, Epstein JI, Humphreys EB, Walsh PC (2010). What are the outcomes of radical prostatectomy for high-risk prostate cancer?. Urology.

[14] Walsh PC. Preservation of sexual function in the surgical treatment of prostatic cancer--an anatomic surgical approach. Important Adv Oncol. 1988;161–170. 3042603

[15] Meng MV, Elkin EP, Latini DM, Duchane J, Carroll PR (2005). Treatment of patients with high risk localized prostate cancer: results from cancer of the prostate strategic urological research endeavor (CaPSURE). J Urol.

[16] Joniau S, Briganti A, Gontero P (2015). Stratification of high-risk prostate cancer into prognostic categories: a European multi-institutional study. Eur Urol.

[17] Fossati N, Passoni NM, Moschini M, et al. Impact of stage migration and practice changes on high-risk prostate cancer: results from patients treated with radical prostatectomy over the last two decades. BJU Int. 2015 10.1111/bju.13125 PMC5160041 25787671

[18] Briganti A, Joniau S, Gontero P (2012). Identifying the best candidate for radical prostatectomy among patients with high-risk prostate cancer. Eur Urol.

[19] Roumiguie M, Beauval JB, Filleron T, et al. External validation of the Briganti nomogram to estimate the probability of specimen-confined disease in patients with high-risk prostate cancer. BJU Int. 2014 10.1111/bju.12763 24684584

