# Supplementary material for: Biochemical recurrence-free survival and pathological outcomes after radical prostatectomy for high-risk prostate cancer
Source: BMC Urol. 2016 Jun 8;16:26. doi: 10.1186/s12894-016-0146-6 (PMC4897874; doi:10.1186/s12894-016-0146-6)
Supplement: Additional file 1: Table S1. — Different groups of risk factors (RF). (DOCX 45 kb) [file 12894_2016_146_MOESM1_ESM.docx]

| Description | | n (%) | Total n (%) |
| --- | --- | --- | --- |
| 1RF | ≥cT2c | 36 (7) | 411 (79.5) |
|  | PSA>20 | 196 (37.9) |  |
|  | GS>7 | 179 (34.6) |  |
| 2RF | ≥cT2c and PSA>20 | 35 (6.8) | 93 (18) |
|  | PSA>20 and GS>7 | 33 (6.4) |  |
|  | ≥cT2c and GS>7 | 25 (4.8) |  |
| 3RF | ≥cT2c and PSA>20 and GS>7 | 13 (2.5) | 13 (2.5) |
