# Supplementary material for: Biochemical recurrence-free survival and pathological outcomes after radical prostatectomy for high-risk prostate cancer
Source: BMC Urol. 2016 Jun 8;16:26. doi: 10.1186/s12894-016-0146-6 (PMC4897874; doi:10.1186/s12894-016-0146-6)

Surgical margin

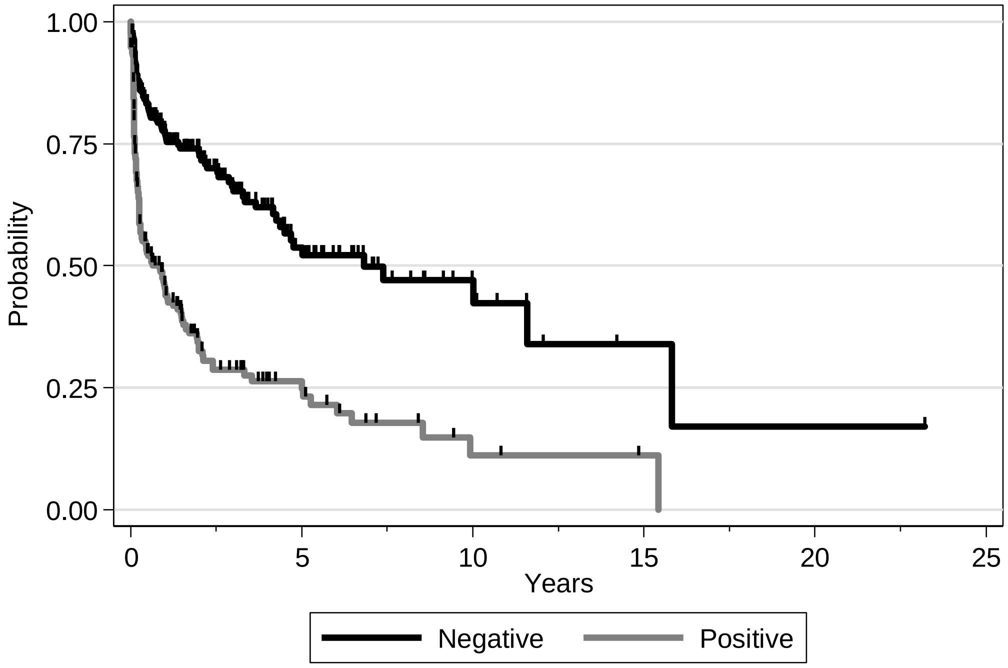

PSA preoperative

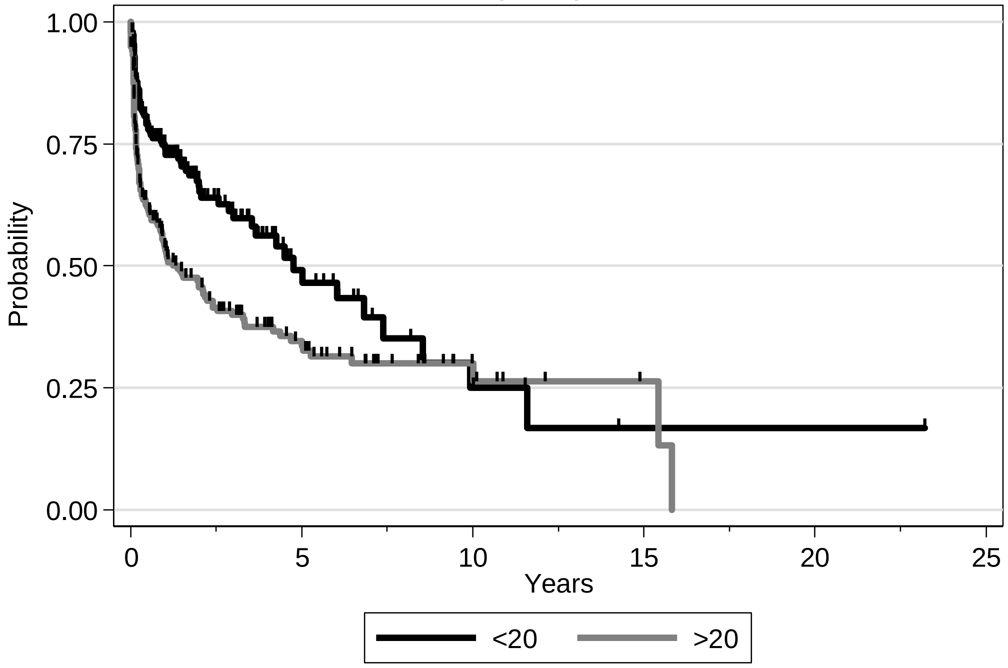

Supplement: Supplementary file 2 [file 12894_2016_146_MOESM2_ESM.docx]
